# Alternative antiviral approaches to combat influenza A virus

**DOI:** 10.1007/s11262-022-01935-3

**Published:** 2022-10-19

**Authors:** Ka Heng Wong, Sunil K. Lal

**Affiliations:** 1 School of Science, Monash University Malaysia, 47500 Bandar Sunway, Selangor DE Malaysia; 2 Tropical Medicine & Biology Multidisciplinary Platform, Monash University Malaysia, Bandar Sunway, 47500 Selangor, Malaysia

**Keywords:** Antigenic drift, Antigenic shift, Antisense oligonucleotides, CRISPR, RNAi, Antiviral

## Abstract

Influenza A (IAV) is a major human respiratory pathogen that contributes to a significant threat to health security, worldwide. Despite vaccinations and previous immunisations through infections, humans can still be infected with influenza several times throughout their lives. This phenomenon is attributed to the antigenic changes of hemagglutinin (HA) and neuraminidase (NA) proteins in IAV via genetic mutation and reassortment, conferring antigenic drift and antigenic shift, respectively. Numerous findings indicate that slow antigenic drift and reassortment-derived antigenic shift exhibited by IAV are key processes that allow IAVs to overcome the previously acquired host immunity, which eventually leads to the annual re-emergence of seasonal influenza and even pandemic influenza, in rare occasions. As a result, current therapeutic options hit a brick wall quickly. As IAV remains a constant threat for new outbreaks worldwide, the underlying processes of genetic changes and alternative antiviral approaches for IAV should be further explored to improve disease management. In the light of the above, this review discusses the characteristics and mechanisms of mutations and reassortments that contribute to IAV’s evolution. We also discuss several alternative RNA-targeting antiviral approaches, namely the CRISPR/Cas13 systems, RNA interference (RNAi), and antisense oligonucleotides (ASO) as potential antiviral approaches against IAV.

## Introduction

Influenza, commonly known as “flu”, is an infectious respiratory disease caused by influenza viruses [1]. These viruses (*Orthomyxoviridae* family) are enveloped, negative-sense single-stranded ribonucleic acid (RNA) viruses with segmented genomes. They can be further classified into four species, influenza A (genus *Alphainfluenzavirus*), B (genus *Betainfluenzavirus*), C (genus *Gammainfluenzavirus*), and D (genus *Deltainfluenzavirus*) viruses [1]. Typically, human influenza is caused by the influenza A and B viruses [1]. To no stranger, the influenza viruses evolve rapidly, producing antigenic diversity via antigenic drift and antigenic shift [2]. Notably, antigenic drift results in the emergence of antigenic variants through various mutations, especially given that the viral RNA-dependent RNA polymerase has high error rates and lack proofreading ability. Meanwhile, antigenic shift contributed to novel strains via random reassortment of genome segments between co-infecting influenza strains [3]. Thus, this leads to seasonal and pandemic influenza when the hemagglutinin (HA) and neuraminidase (NA) proteins in IAV mutate sufficiently to allow immune evasion. This rapid pace of pace renders the acquired immunity through vaccinations and prior infection ineffective towards the new variants that emerge from antigenic drift and shift [4].

Typically, human influenza epidemics occur during winter months in temperate regions but are prevalent at any time of the year for regions near the equator [2]. The symptoms may range from mild upper respiratory disease with fever, sore throat, cough, muscle pain, fatigue, headache, and weakness to severe secondary bacterial infection of the lower respiratory tract or lethal pneumonia [1]. In 2019, up to 200,000 deaths worldwide were caused by influenza [5]. In particular, IAV contributes to influenza pandemics and further aggravates the spread of novel IAV strains globally, tempering the lives of many [1, 2]. Notably, the 1918 ‘Spanish flu’ pandemic, also termed the ‘greatest medical holocaust in history’, has led to approximately 40 to 50 million deaths [6]. This was succeeded by the ‘1957 Asian flu’, ‘1968 Hong Kong flu’, and the 2009 ‘Swine flu’, which caused at least 500,000 deaths in each outbreak [6]. Consequently, economic and social activities were severely impacted [6]. Additionally, the emergence of genetic variability rendered treatments ineffective against drug-resistant influenza strains, which has especially prominent and severe effects amongst immunocompromised individuals [3, 7]. Currently, the US Food and Drug Administration (FDA) has approved antivirals that target NA (zanamivir, oseltamivir) and matrix protein 2 (M2) ion channels (rimantadine, amantadine) [1]. NA inhibitor (NAI) drugs are currently the drug of choice against influenza infection, as amantadine resistance is already highly prevalent [3].

Due to the devastating consequences of pandemic influenza and annual epidemic outbreaks, it is crucial to monitor and study the characteristics and mechanisms involved in genetic changes in IAV. Moreover, the constant mutation of influenza proteins reduces therapeutic efficacy, further limiting treatment options. Therefore, alternative antiviral approaches for influenza treatment should be explored to benefit public health and manage potential future pandemics better by thoroughly understanding risks associated with influenza evolution. In this review, we aim to elucidate the characteristics and underlying mechanisms contributing to antigenic changes of IAV. Several potential emerging antiviral approaches against IAV will also be addressed in this context.

## IAV characteristics

### Significance of HA & NA

The homotrimer HA facilitates viral entry by binding to host cell surface receptors containing sialic acid (SA) [1, 2]. The HA precursor (HA0) consists of two subunits, namely the HA1 receptor-binding domain and HA2 fusion peptide [8]. Upon successful binding, the virus are internalised into an endosome via receptor-mediated endocytosis. The low-pH environment of endosomes subsequently triggers the conformational changes in HA0, causing HA1 to be maintained but exposing HA2 [8]. When HA2 integrates into the endosomal membrane, viral and endosomal membranes fuse as it comes into contact, followed by the opening of matrix protein 2 (M2) ion channels (selective proton-ion channel) which acidifies the viral core. The acidification leads to the release of viral ribonucleoprotein (vRNP) from the matrix 1 protein (M1) scaffold and into the host cytoplasm [8]. Notably, the HA binding is one of the determinants of host specificity; avian and equine influenza HA preferentially binds to α-2,3-SA receptors, whilst human-adapted influenza prefers α-2,6-SA receptors [9]. As swine possess both receptors, it is a suitable “mixing vessel” that may create novel reassortant IAV strains [9]. Furthermore, the pH stability of HA determines viral tropism as endosomal pH varies between species [10]. Viral tropism is defined as an infection of specific cells, tissues, or hosts effectively by a given virus, which can impact outcomes of infection in the host [11]. For example, the HA of seasonal influenza viruses prefers a pH of 5.0, whilst highly pathogenic avian influenza (HPAI) viruses HA works optimally at pH 5.3–5.6 [10]. HA may undergo denaturation at suboptimal pH, leading to the loss of function, eventually obstructing membrane fusion and genome release [1]. Hence, the importance of HA in the IAV life cycle cannot be overstated.

Meanwhile, NA is involved in the viral release and assisting viral spread [1]. This occurs by the cleavage of SA from glycolipids and glycoproteins present on infected host cells, allowing the new virions to bud out from the infected cells and readily infect other cells of individuals [12]. In cases of defective NA, HA of progeny virions may bind to SA glycoproteins of infected cells, forming aggregates on cell surfaces that inhibit viral release [13]. The above mentioned forms the concept of NAI antivirals in influenza treatment and emphasises the importance of NA in the IAV life cycle.

## Underlying mechanisms and outcome of antigenic changes

Influenza viruses are well known for their rapid evolution [2]. This was mainly attributed to the high mutation rates of IAV and a short generation time, allowing for rapid propagation [2]. Notably, the generation time is the time required for infection to spread within a population, which is approximately 2.5 days for influenza viruses [14, 15]. In addition, mutations conferring immune escape are positively selected and passed down to progenies, which are then distributed rapidly and widely [2]. Moreover, the (1) lack of proofreading ability of the viral RdRp and (2) segmented nature of the viral genome-mediated virus evolution facilitate genetic changes by the gradual accumulation of mutations and genome reassortment [1].

### Mutation

The emergence of antigenic variants or “strains” is typically contributed by the continuous accretions of nucleotide mutations and amino acid substitutions in influenza surface antigens, especially HA [16]. Wrong nucleotides are integrated at rates of 10^−3^ to 10^−4^ during viral replication and remain in the viral genome due to the viral RdRp [2]. Thus, substantial mutations in the genes encoding for HA and NA may be passed down during the replicative cycle, causing the new progeny virus to differ significantly from the original strain [17]. This was proven by the affliction of flu several times throughout a human lifespan and the annual re-emergence of seasonal influenza [17]. Hence, vaccine strains must be constantly updated to match current circulating strains to better control seasonal influenza.

Other than that, amino acid substitution mutations in antigenic regions of HA may contribute to altered host receptor preferences [2]. Studies have demonstrated that G186V mutation amongst avian H7N9 strains caused adaptation towards human host receptors [18, 19] (Table 1). Another study has shown that Gln226Leu in avian H7N9 contributed to enhanced human receptor binding [19]. This Gln226Leu mutation was also found in the 1957 H2 and 1968 H3 pandemic strains [19]. However, a stronger preference for avian receptors remained according to both studies. This indicated that more extensive mutations are required to adapt to new hosts. Furthermore, a combination of K58I and G219S mutations in HA proteins of avian A/Anhui/1/13 (H7N9) virus has demonstrated increased affinity to human SA receptor [20]. Although antigenic changes may alter receptor preferences, they generally do not lead to pandemics, albeit causing zoonotic influenza that may affect humans [2]. On a side note, extensive studies on bat-associated influenza viruses should be performed as IAV transmission between mammals may require less extensive mutations for human adaptation.

**Table 1:** Summary of amino acid substitution, abbreviation, and outcome of mutations in IAV

Amino acid substitution mutation	Abbreviation	Outcome	Study
Trades glycine (G) for valine (V) at position 186 in HA	G186V	Adaptation of avian H7N9 virus to human α-2,6 SA receptors	[18, 19]
Trades glutamine (Q) for leucine (L) at position 226	Gln226Leu or Q226L		[19]
Trades lysine (K) for isoleucine (I) at position 58	K58I	Increased affinity of H7N9 to α-2,3 and α-2,6-linked SA receptor	[20]
Trades glycine (G) for serine (S) at position 219 in HA	G219S		
Arginine substitution at position 292 of NA protein	R292	Contributes to NAI resistance	[23–25]
Asparagine substitution at position 294 of NA protein	N294		
Isoleucine substitution at position 122 of NA protein	I122		
Glutamic acid substitution at position 119 of NA protein	E119		
Aspartic acid substitution at position 198 of NA protein	D198		
Histidine substitution at position 274 of NA protein	H274		
Trades histidine (H) for tyrosine (Y) at position 274 of NA protein	H274Y	Most common mutation contributing to NAI resistance Associated with reduced viral fitness	[24]
Trades valine (V) for isoleucine (I) at position 274 of NA protein	V241I	Secondary mutation alongside H274Y that offset reduced viral fitness	[23, 26, 28]
Trades Asparagine (N) for lysine (K) at position 274 of NA protein	N369K		

Unlike the H9N2 bat viruses, the recently discovered bat influenza viruses (H17, H18) do not bind to α-2,3-linked SA receptors but to the major histocompatibility complex class II (MHC-II) instead for host cell entry [21, 22]. Since MHC-II molecules are found in many different animals, such as humans, pigs, and chickens, this may give a broad host range for IAV infection [21]. Unravelling the underlying mechanisms of MHC for IAV infection in the host may provide insight into the adaptations required for IAV transmission between mammalian hosts’ species. This is beneficial for the monitoring of potential zoonotic influenza outbreaks. Hence, further study on MHC-II as receptors for cell entry by bat viruses should not be overlooked.

Furthermore, genetic mutations may also contribute to NAI resistance in IAV. NAI are sialic acid analogues that block NA active sites and prevent NA activation, thereby preventing viral release [23]. Studies have found that amino acid substitution mutations at R292, N294, I122, E119, D198, and H274 (in N2 subtypes, H275 in N1 subtypes) of NA contribute to decreased NAI susceptibility [23–25]. Akin to antibiotic-resistant bacteria, the antiviral usage may bring up selection pressure within resistant variants. Notably, the H274Y mutation was commonly found to confer NAI resistance accompanied by a decrease in viral fitness in IAV [23, 24, 26]. Viral fitness is denoted as the virus’s ability to replicate and give rise to infectious progeny in a specific environment [27]. Whilst locations of the mutations may be a deciding factor, further study is required to fully unravel the underlying mechanisms. However, other studies have demonstrated NAI resistance mutations in IAV without compromising viral fitness. A study found that secondary NA mutations such as V241I and N369K granted robust viral fitness on H275Y-mutant H1N1 viruses in ferrets [24]. It had increased expression and enzymatic activity of viruses, which improved the viral fitness and allowed sustained transmission compared to H275Y-mutant H1N1 viruses without secondary mutations [24]. Similarly, a study also demonstrated enhanced viral fitness in H1N1 pandemic viruses with V241I and N369K mutations in ferret models, which had higher virus titres than H275Y mutants without secondary mutations [28]. The improved viral fitness facilitates transmission of IAV with reduced NAI susceptibility, posing enormous therapeutic challenges and therefore should be monitored closely.

### Reassortment

The segmented RNA genome of influenza facilitates genome reassortment, which can bring forth new genetic traits [29]. As mentioned above, reassortment involves the exchange of segmented genomes during co-infection of two or more influenza strains [29]. New antigenic patterns may arise, but reassortment may also cause significant changes in genotype and phenotype of progeny viruses in a process known as genetic shift [29]. Consequently, new IAV subtypes may emerge, causing influenza pandemics, especially in animal influenza viruses that become capable of infecting humans [1]. For instance, co-infection of the avian and human IAV within a host or vessel may facilitate the reassortment of genes between the two strains, producing a novel reassorted IAV strain that acquired genes from both viruses [8, 30]. Therefore, the minimal pre-existed immunity against the novel IAV within the human population confers rapid spread within the community.

IAV strains have caused influenza pandemics in 1918, 1957, 1968, and 2009 involving reassortment between human IAV and other host species in the past century. Since antibodies against influenza virus antigens are only elicited from prior exposure, it is ineffective towards new variants [4]. It was found that reassortment caused emergence of 1918 IAV pandemic strain by converging avian N1 (and other internal genes) and human H1 subtype [31]. Notably, the internal genes originated from equine H7N7 lineage, which was also introduced into the avian strain by reassortment [31]. Interestingly, the HA and NA of the 1957 H2N2 pandemic strain plus HA of 1968 H3N2 pandemic strain were suggested to originate from avian influenza [2]. Furthermore, reassortment of internal genes and HA/NA proteins between avian, human, and swine IAV caused emergence of the 2009 H1N1 pandemic strain. It comprised avian influenza viruses (North America), human H3N2, and H1N1 and H1N2 swine influenza (European and North American, respectively) [2]. Overall, evidence showed that reassortment between IAV facilitates the genetic exchange that contributed to the emergence of novel IAVs that caused pandemic influenza.

Furthermore, animal IAVs such as avian H5N1, avian H7N9, and swine H3N2 were found to infect humans occasionally [1]. These infections are more prevalent amongst populations in close contact with infected poultry and swine, suggesting that zoonotic pandemic influenza may emerge from these regions and should be monitored [1]. However, these cases are isolated and were not reported to cause human-to-human transmission [1]. Thus, this postulates the need for further adaptation by zoonotic influenza viruses to become transmissible between humans. Moreover, reassortment may compromise viral fitness of progenies, affecting the virus transmission and pathogenicity [29, 32]. However, the requirements and feasibility of the adaptations are yet to be fully understood [1]. Predictions for future pandemics remain challenging since genomic reassortment may only be traced retrospectively via gene analysis. Additionally, the difficulty in determining adaptation to new hosts by genome reassortment adds a considerable challenge. Therefore, it is vital to explore and develop alternative antiviral options to combat IAV.

## RNA-targeting approaches as alternative antivirals

Alternative approaches are required as antigenic changes may cause IAV strains to resist current therapeutic programmes [7]. Furthermore, there is an increasing demand for new antivirals as current practices may be insufficient for a future pandemic. RNA-targeting approaches such as RNA interference (RNAi), antisense oligonucleotides (ASO), and clustered regularly interspaced short palindromic repeat (CRISPR)/CRISPR-associated protein (Cas) system technologies may provide gene-specific therapy against IAV infections, which will be further explored in this section.

### RNAi

RNAi causes sequence-specific suppression of gene expression via mRNA degradation [33]. It is triggered by double-stranded RNA known as short interfering RNA (siRNA) (21 to 25 nucleotides long), with base pairing and 2-nucleotide overhangs at 3’ end [7].

**Fig. 1:**
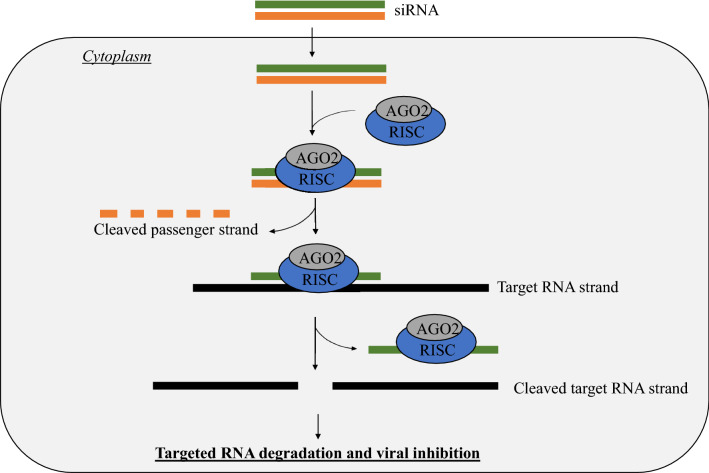
Schematic diagram showing RNAi-mediated gene knockdown. Artificial siRNA is introduced into the host cells and subsequently binds to the RISC complex in the cytoplasm. The passenger strand is degraded by the helicase activity of the RISC complex, leaving behind the guide siRNA strand that directs targeted RNA cleavage and degradation. Notably, the AGO2 protein is responsible for catalysing the cleavage process. Hence, this disrupts the flow of genetic information for the viral protein, eventually causing viral inhibition

Gene silencing by RNAi involves dicers in nature, which are endonucleases that cleave double-stranded RNA into siRNA [34]. The siRNA will be incorporated into the RNA-induced silencing (RISC) complex, which identifies complementary mRNA according to Watson–Crick base pairing and subsequently guides mRNA degradation. The ribonuclease, which is the protein argonaute-2 (AGO2), is part of the RISC complex that catalyses the cleavage of target RNA [34] (Table 2). Alternatively, siRNA may be artificially introduced to cells via nanoparticles. Cellular uptake occurs by encapsulation into endosomes and subsequent release of siRNA into the cell cytoplasm following endosomal escape [34]. Similarly, the siRNA will be incorporated into the RISC to guide mRNA degradation as seen in Fig. 1 [34]. The mRNA degradation disrupts the flow of genetic information, inhibiting the production of functional proteins.

**Table 2:** Summary of similarities and differences between various RNA-targeting approaches for IAV

RNA-targeting approaches	Differences	Similarities
RNAi	●siRNA is dsRNA that guides targeted mRNA degradation ●Degradation of viral RNA is performed by the AGO2 RNase of the RISC complex	●Targets conserved regions of the viral RNA for gene silencing ●Utilises a guide RNA and RNase as effector molecules ●Guide RNA has complementary sequences to the target RNA, which is used to identify the target sequences for sequence-specific degradation
ASO	●ASO are ssDNA that guides targeted vRNA degradation ●Degradation of viral RNA is done by the RNase H endonuclease	
CRISPR/Cas13	●crRNA are RNA molecules that guide target viral positive-sense RNA degradation ●Degradation of viral RNA is done by Cas13 endonuclease

Conserved mRNA regions should be targeted for effective RNAi due to the highly mutable influenza genome [7]. Thus, genes encoding HA and NA should be excluded due to tendencies for mutations [35]. Moreover, secondary structures of target mRNA, such as hairpin structures, could impact siRNA efficiency. Therefore, targeting conserved structural motifs with accessible regions in viral RNA may provide better inhibitory outcomes [7, 33]. In IAV, all viral genes are essential in viral replication [35]. An early study designed multiple siRNA against NP, PA, PB1, PB2, NS, and M mRNA demonstrated potent H1N1 inhibition by anti-NP and anti-PA siRNAs in Madin–Darby Canine Kidney (MDCK) cells [33]. In the same study, the H1N1-infected cells transfected with NP-1496, the siRNA which targeting NP sequence beginning at position 1496, displayed only a slight increase in virus titre over time compared to mock control treatment at 20-, 30-, 40-, 50-, and 60-h post-transfection, suggesting good viral inhibitory effect.

On the other hand, the in vivo assay of the same study demonstrated a decreased virus titre in allantoic fluid of 10-day-old embryonated chicken eggs at 17 h after injection with different siRNA (2.5 nmol), 500 PFU of PR8 virus, and oligofectamine [33]. Amongst the various siRNAs used, the NP-1496, PB1-2257, and PA-2087 potently inhibited IAV viral replication in vivo [33]. Another study also demonstrated siRNA613 and siRNA682 targeting structurally conserved and oligonucleotide-accessible locations of mRNA segment 5 (regions 613–631 and 682–700, respectively) significantly reduced viral copy number to 14.7% and 22.0%, respectively, in MDCK cells as compared to the negative control (non-targeting siRNA) [7]. This scenario may be due to NP being highly involved in viral replication [36], more abundant than other viral proteins [37], highly conserved amongst IAV strains, and less prone to antiviral resistance [7]. As shown above, the NP-1496 siRNA suppressed viral replication with minimal increment in viral load post-siRNA transfection, further emphasising great antiviral potential [33]. On the other hand, the siRNA NP-231 that targets NP sequences at position 231 did not cause a substantial reduction in virus titres [33]. This showed that different target sequences might contain structural motifs that may hinder RNAi efficacy of siRNA, despite also targeting conserved gene sequences of viral proteins. Another study used various anti-M2 siRNA (M950, M747, M832) that targeted different structural regions of M2 mRNA of H1N1 and demonstrated different levels of inhibition (54.7%, 30.6%, and 48.9% matrix RNA reduction, respectively), further affirms the different efficacies at different target sequences in the conserved regions [38]. Although viral inhibition by anti-M siRNA is lower than anti-NP siRNA, it caused a statistically significant reduction in viral load [38]. Despite that, cytotoxicity studies were not performed. Possible side effects with risk–benefit balance should be assessed and further evaluated for safety usage. Overall, the RNAi therapy demonstrated promising results, with specific viral targets producing more substantial viral inhibitory outcomes.

Apart from that, the siRNA may be administered by inhalation for clinical therapy and prophylaxis purpose against IAV infection [35]. As influenza viruses infect the upper respiratory tract, siRNA may be administered with nebulizers, effectively carrying antiviral agents directly to the site of infection. Thus, nebulisers may provide a quick and effective in vivo delivery over intravenous injection, which requires polymer carriers or liposomal formulations (lipid-bilayer vesicles) to facilitate cellular delivery and uptake (endosomal fusion) [7, 39]. Moreover, polymers such as polyethylenimine (PEI) may also be used to facilitate the uptake of intravenously administered siRNA into lung tissues [7]. However, these polymers may trigger host immune reactions, causing non-desirable inflammation and offsetting the therapeutic goals [35]. Hence, effective and safe siRNA drug delivery methods should be further evaluated for an effective clinical antiviral treatment.

### Antisense oligonucleotides (ASO)

**Fig. 2:**
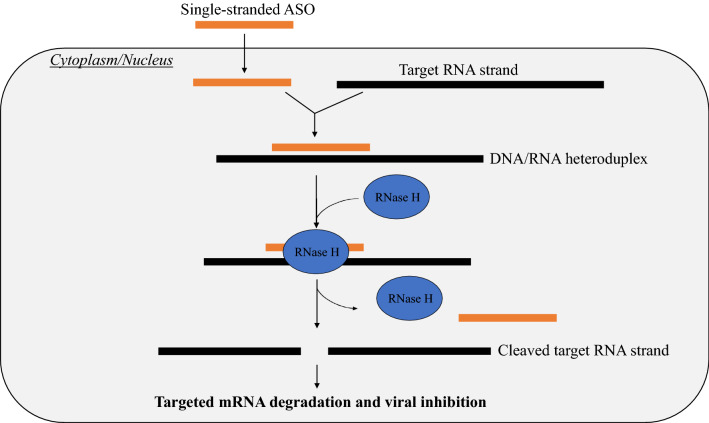
Schematic diagram showing the mechanism of action for ASO-mediated gene knockdown. The introduced ASOs bind to the target viral RNA strand through their complementary sequences, leading to the formation of a DNA/RNA heteroduplex. When a stretch of > 5 nucleotides pairs between the ASO and target strand, the RNase H enzyme is activated to cleave the target RNA strand. Due to this, it leads to targeted viral mRNA cleavage and degradation. Hence, the expression of the target viral protein reduces and eventually leads to viral inhibition

ASO are short, single-stranded DNA oligonucleotides (3’–5’ direction) complementary to mRNA targets (5’–3’) used in sequence-specific suppression of gene expression [40]. Akin to siRNA, ASOs binds to complementary target sequences by Watson–Crick base pairing. After ASO enters infected host cells and bind target viral mRNA containing a complementary sequence, this leads to the formation of RNA–DNA heteroduplex. When a stretch of > 5 nucleotides pairs between the ASO and target gene, the ribonuclease H (RNase H) present within host cells are activated to cleave the heteroduplex as seen in Fig. 2, leading to a reduction in target gene translation and causing downregulation of gene expression [41]. This process may take place in the nucleus or the cytoplasm as ASO was shown to accumulate in these locations [42]. Certain conserved structural motifs are also targeted as RNA secondary structure impacts protein function [42]. By disrupting structural motifs, potent inhibition of viral replication may be achieved.

Currently, only secondary structures for vRNA segments 5, 7, and 8 of IAV have been mapped and utilised in designing experimental anti-influenza ASO. The vRNA segments were screened for conserved structural motifs via bioinformatics tools, and several ASOs were created based on the predicted self-folding of vRNAs [37, 42]. A recent study targeting mRNA segment 5 (encoding NP) with ASO showed approximately 43–83% reduction in virus replication in infected MDCK cells [37]. Notably, ASO 883-11L targeting 878–888 nucleotide regions was found to be the most effective (83%). Similarly, amongst the five best ASOs that target IAV segments 7 and 8 (encoding matrix proteins and NSP, respectively) have caused a five to 25-fold virus inhibition in MDCK cells [42]. Moreover, a study showed that 750 nM of ASO 613 and ASO 682, targeting regions 613–631 and 682–700, respectively, reduced viral RNA copy number by 26.9% and 26.8% in genome segment 5 [7].

Furthermore, ASO with locked oligonucleotides (LNA) for improved duplex stability showed significantly higher inhibition (P < 0.05) compared to ASOs without LNA substitution. For example, a 2.5- to tenfold higher inhibition was observed by ASO 68-11L (LNA modified) compared to ASO68-11 M (LNA unmodified) [42]. In addition, ASO276-20L (LNA-modified) demonstrated a statistically significant threefold greater inhibition than the unmodified ASO276-20, which did not cause significant viral inhibition [42]. Similarly, the LNA-modified ASO, ASO883-11L, inhibited influenza replication by 88% and was fivefold more effective than the non-LNA-modified variant, ASO883-11 M, in MDCK cells [37]. This was because unmodified ASO is susceptible to degradation by nucleases, which reduces the antiviral efficacy of ASOs [41]. Hence, with locked oligonucleotides fortified formulation, ASO demonstrated great potential in inhibiting influenza replication in vitro.

Unmodified ASO is susceptible to degradation by nucleases, which targets phosphodiester backbones of nucleic acids which may reduce antiviral efficacy of ASOs [41]. Thus, oligonucleotide stability may be fortified via chemical modification to the nucleotide backbone. For instance, phosphorothioate (PS) modification replaces non-bridging oxygen atoms with sulphur within the phosphodiester backbone to improve drug bioavailability, which is commonly used to improve ASO stability without impacting RNaseH activity and ASO solubility [41]. However, PS may cause non-specific interactions with plasma, intracellular, and cell surface host proteins that may lead to cellular toxicity [43]. Thus, RNaseH may be induced to target host proteins which may cause unintended harm. Based on the studies above, PS ASOs were not explored. Despite that, studies have shown good safety profiles with minimal cytotoxicity for ASOs (unmodified and LNA-modified) in MDCK cells post-treatment [37, 42]. Therefore, further evaluation of ASOs and its modifications should be performed to improve the influenza virus load reduction and manage cell cytotoxicity stop the cellular uptake, thus maximising the potential of ASOs against the IAV infection.

### CRISPR/Cas13

**Fig. 3:**
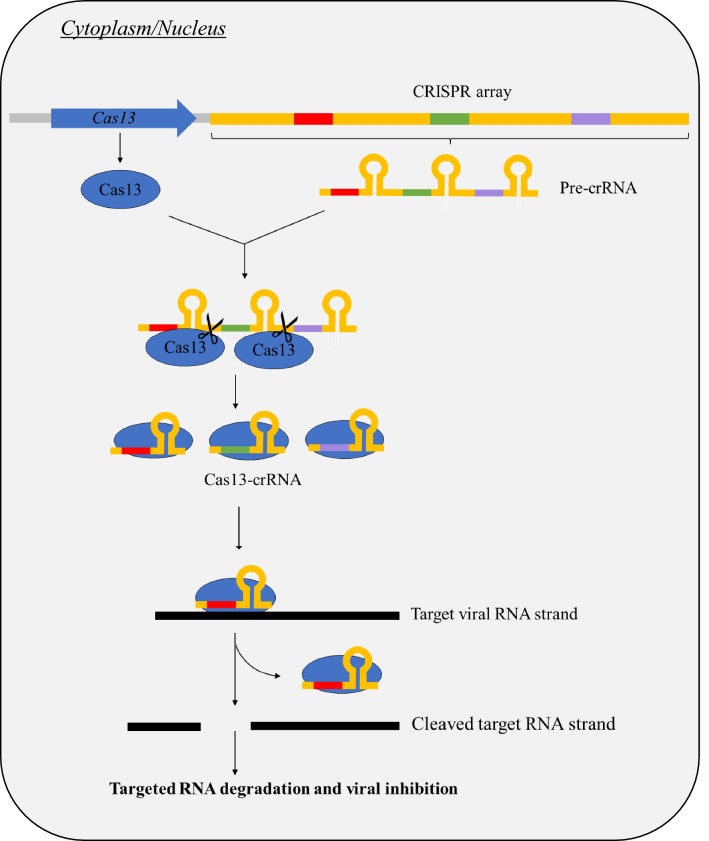
Schematic diagram for CRISPR/Cas13 system in targeted viral RNA knockdown. In cells introduced with the CRISPR/Cas13 system, the Cas13 gene and the CRISPR array are transcribed to form the Cas13 endonuclease enzyme and pre-crRNA, respectively. This pre-crRNA will be processed into mature crRNA by the Cas13 enzyme and subsequently associates with the enzyme to form the Cas13-crRNA complex. When the crRNA strand within the complex identifies complementary sequences on the target viral RNA strands, this triggers the cleavage and subsequent degradation of the strands. Thus, virus inhibition may be achieved due to the disrupted viral protein synthesis

CRISPR/Cas systems were originally a bacterial defence system against bacteriophages and foreign nucleic acids [44]. Recent studies have utilised CRISPR/Cas13 systems in RNA knockdown in mammalian and plant cells, suggesting roles as a potential antiviral [45]. In CRISPR/Cas13, a single-guide RNA (gRNA) identifies specific gene targets which are subsequently cleaved by the Cas13 enzyme (effector) as seen in Fig. 3. The gRNA is also known as CRISPR-associated RNA (crRNA), which are customized gene sequences complementary to the target gene [44]. Thus, Cas13 may be programmed to target specific regions of the viral genome by creating crRNAs that complement the targeted regions of the viral genome. With the viral RNA being used as a template, RNA degradation via CRISPR/Cas13 system may be utilised to prevent the production of the essential viral proteins and therefore prevent viral replication [44]. There are four types of Cas13 enzymes (A-D), in which Cas13a/b/c have already demonstrated high efficacy and specificity of RNA knockdown in mammalian cells [45]. These effectors utilise a protospacer flanking sequence (PFS) to cleave RNA targets effectively [45]. Despite the novelty of Cas13d effectors, it has demonstrated efficient RNA knockdown in mammalian cells. Furthermore, Cas13d are PFS independent, which may improve its overall feasibility. Hence, CRISPR effectors bring great potential as an antiviral approach.

Recent studies performed successful CRISPR/Cas13 RNA knockdown in IAV, which reduced virus load in mammalian cells. A study demonstrated IAV viral RNA degradation in infected adenocarcinoma human alveolar basal epithelial cells (A549) via Cas13d [46]. Initially, A549 cell lines expressing Cas13d were created before transfecting with different crRNAs that target conserved regions of various IAV genome segments. Specifically, conserved regions involved in viral packaging were targeted as it could potentially inhibit a broad range of IAV strains amongst which the cRNAs targeting segment 4 and segment 6, respectively, showed significant viral inhibition [46]. At MOI of 0.5, IAV was inhibited by 59% and 78% by crRNAs targeting segment 4 and 6, respectively. Moreover, another study has successfully reduced IAV RNA load in infected MDCK cells with Cas13b [44]. Five different crRNAs were designed to target the mRNA and cRNA of M and NP proteins (namely the M1, NP1, NP2, NP3, and NP4 crRNAs, respectively), causing a seven to 22-fold reduction, in which anti-M1 crRNA caused the most significant virus reduction in MDCK cells. However, A549 cells may reflect outcome of CRISPR/Cas13 in humans more accurately compared to MDCK, as human influenza primarily affects the human respiratory system. Apart from that, it was found that the localisation of Cas13b in the nucleus or cytoplasm of mammalian cells impacted crRNA activity. Anti-NP1 crRNA greatly reduced IAV RNA levels when Cas13b was localised to cytoplasm, but this reduction was absent when Cas13b was localised to the nucleus [44]. Since influenza virus replication occurs in the nucleus [1], it is suggested that Cas13b functioned before entry of vRNP to host nucleus or before assembly of new virus particles. Hence, localization of Cas13b to the host nucleus may improve inhibition efficacy.

However, CRISPR/Cas systems face limitations in safe and effective in vivo delivery methods [46]. Although lipid nanoparticles and chemical polymers have been suggested to introduce associated crRNA and Cas13 RNA to host cells, components may be difficult to control unlike conventional drugs that deliver a measured dosage of active ingredients with known degradation kinetics and bioavailability. Besides that, the Cas13 effectors must be localised to human respiratory cells, which is the influenza infection site, to ensure efficient viral inhibition [46]. This further contributes to the list of challenges [46]. Thus, previous studies on siRNA and ASO or other diseases may be referenced in directing future studies on in vivo delivery, such as utilising lipid nanoparticles, chemical polymers, and peptide particles. For example, self-assembled peptide–poloxamine nanoparticles have been used in delivering nucleic acids to the respiratory system by nebulizers or nasal spray for gene therapy optimised against cystic fibrosis [46]. Hence, further optimization for clinical administration is required to establish the safety and efficacy of CRISPR effectors as influenza antiviral agents.

## Conclusion

IAV is prone to antigenic changes due to accumulation of genetic mutations and genome reassortment involving the HA and NA viral proteins, which contributes to antigenic drift and antigenic shift. Since host immunity may be elicited from antigenic exposure during vaccination or infection, significant antigenic changes in IAV allow for immune evasion and alterations in host receptor preference, disrupting the host immune protection system. Thus, slow antigenic drift and reassortment-derived antigenic shift contribute to the annual re-emergence of seasonal influenza and, occasionally, pandemic influenza, which pose a significant threat to global healthcare. Moreover, genetic changes may contribute to NAI resistance without compromising viral fitness and further restrict already limited therapeutic options. Findings show that ASO, RNAi, and CRISPR/Cas13 showed promising results both in vitro and in vivo for viral gene silencing. Additionally, CRISPR-Cas13 systems may demonstrate the highest antiviral potential. As RNAi and ASO present cytotoxicity and safety concerns, CRISPR-Cas13 systems allow precise and gene-specific therapy with great potential for personalised medicine. However, further optimization in terms of safety, bioavailability, and delivery methods are still required.
